# Host Determinants of MERS-CoV Transmission and Pathogenesis

**DOI:** 10.3390/v11030280

**Published:** 2019-03-19

**Authors:** W. Widagdo, Syriam Sooksawasdi Na Ayudhya, Gadissa B. Hundie, Bart L. Haagmans

**Affiliations:** Department of Viroscience, Erasmus Medical Center, 3025 Rotterdam, The Netherlands; w.widagdo@erasmusmc.nl (W.W.); s.sooksawasdi@erasmusmc.nl (S.S.N.A.); g.hundie@erasmusmc.nl (G.B.H.)

**Keywords:** MERS-CoV, transmission, pathogenesis, host factors, DPP4

## Abstract

Middle East respiratory syndrome coronavirus (MERS-CoV) is a zoonotic pathogen that causes respiratory infection in humans, ranging from asymptomatic to severe pneumonia. In dromedary camels, the virus only causes a mild infection but it spreads efficiently between animals. Differences in the behavior of the virus observed between individuals, as well as between humans and dromedary camels, highlight the role of host factors in MERS-CoV pathogenesis and transmission. One of these host factors, the MERS-CoV receptor dipeptidyl peptidase-4 (DPP4), may be a critical determinant because it is variably expressed in MERS-CoV-susceptible species as well as in humans. This could partially explain inter- and intraspecies differences in the tropism, pathogenesis, and transmissibility of MERS-CoV. In this review, we explore the role of DPP4 and other host factors in MERS-CoV transmission and pathogenesis—such as sialic acids, host proteases, and interferons. Further characterization of these host determinants may potentially offer novel insights to develop intervention strategies to tackle ongoing outbreaks.

## 1. Introduction

Middle East respiratory syndrome coronavirus (MERS-CoV) is a novel pathogen that was isolated in late 2012 [1]. Since then, the virus has caused multiple outbreaks and infected more than 2000 individuals, [2] who then develop a respiratory infection ranging in severity from asymptomatic to fatal [3,4]. Severe-to-fatal MERS-CoV patients have a higher chance of transmitting this virus since they shed a higher amount of virus progeny in comparison to the asymptomatic-to-mild ones [5,6,7,8]. Identifying and quarantining these patients in healthcare facilities where outbreaks have occurred, together with implementing proper infection control, has been effective in reducing transmission and containing these outbreaks [9,10]. However, new MERS-CoV cases are still being reported, especially in the Arabian Peninsula [2,11]. This is partly due to the continuous zoonotic introduction of this virus to the human population in this region by dromedaries [12]. The dromedary camel is the only animal species that has been reported to transmit this virus to humans [13,14,15,16]. MERS-CoV infection in these animals merely causes mild upper respiratory tract infection [17,18], but seroepidemiological studies showed that this virus has been circulating in dromedary camels for decades, suggesting the efficient transmission of MERS-CoV in this species [19,20,21,22].

Although the clinical manifestations, as well as transmission, are remarkably different in MERS-CoV-infected humans and dromedary camels, the viruses isolated from these two species are highly similar, if not indistinguishable [12,16]. This indicates that host factors play a significant role in MERS-CoV pathogenesis and transmission. However, the identity of these host factors and how they affect the pathogenesis and transmission of MERS-CoV are generally not well understood. Dipeptidyl peptidase-4 (DPP4)—the MERS-CoV receptor, sialic acids, proteases, and interferons are all examples of potentially critical host factors that have been shown to affect MERS-CoV infection in vitro [23,24,25,26]. This review highlights the role of some MERS-CoV-interacting host factors—especially DPP4—in MERS-CoV pathogenesis and transmission.

## 2. MERS-CoV-Interacting Host Factors

**Figure 1:**
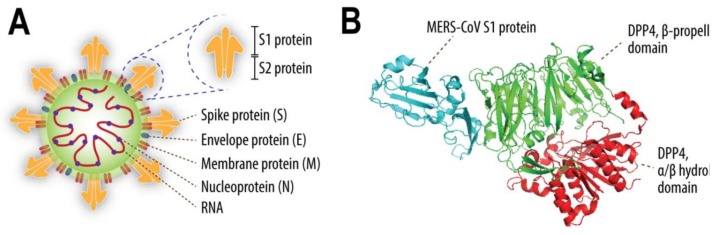
Schematic figure depicting four structural proteins of Middle East respiratory syndrome coronavirus (MERS-CoV), i.e., S, E, M, and N proteins (**A**); a cartoon representation of MERS-CoV S1 protein binding to DPP4 (PDB code 4L72) (**B**). The S protein consists of the S1 and S2 subunits. The α/β hydrolase domain of DPP4 is indicated in red, β-propeller domain in green, while part of the MERS-CoV S1 protein is shown in blue.

MERS-CoV infection of a target cell is initiated by the virus attachment to the cell surface [23,27]. MERS-CoV uses the N-terminal part of its spike (S)—the so called S1 protein (Figure 1A)—to bind to two host cell surface molecules, dipeptidyl peptidase-4 (DPP4) and α2,3-sialic acids [23,24]. DPP4 is the functional receptor of MERS-CoV; its absence renders cells resistant to this virus, while its transient expression in non-susceptible cells permits viral replication [23]. DPP4 is a serine exopeptidase, which is either expressed at the cell surface or shed in a soluble form. It has the capacity to cleave-off dipeptides from polypeptides with either l-proline or l-alanine at the penultimate position. Accordingly, DPP4 is capable of cutting various substrates, such as hormones, cytokines, chemokines, and neuropeptides, allowing it to be involved in multiple physiological functions as well as pathophysiological conditions [28]. This enzymatic activity is mediated by the α/β hydrolase domain of DPP4, while MERS-CoV infection is mediated by the binding of S1 protein to the β-propeller domain of this exopeptidase (Figure 1B) [28,29,30,31]. There are 11 critical residues within the β-propeller domain that directly interact with the S1 protein [29,30,31]. These residues are quite conserved in camelids, primates, and rabbits—species shown to be susceptible to MERS-CoV [17,31,32,33]. In contrast, ferrets, rats, and mice resist MERS-CoV infection due to differences in some critical DPP4 residues [31,34,35,36]. These data illustrate that DPP4 has the capacity to determine the host range of MERS-CoV.

Other MERS-CoV-interacting host factors besides DPP4 are less extensively studied and have mostly been investigated in vitro. Glycotopes of α2,3-sialic acids coupled with 5-N-acetylated neuraminic acid are recognized by the S1 protein of MERS-CoV during attachment [24]. In the absence of these glycotopes, MERS-CoV entry is reduced but not abolished, indicating their function as an attachment factor rather than a receptor [24]. Besides α2,3-sialic acids, CEACAM5 and GRP78 have also been suggested to be attachment factors for MERS-CoV, but their roles in vivo during MERS-CoV infection are not clear at this moment [37,38]. Post attachment, MERS-CoV uses the C-terminal part of its S protein—known as S2 (Figure 1A)—to interact with host proteases, such as furin, TMPRSS2, and cathepsins [39,40,41,42]. These proteases cleave the S protein and induce conformational changes, allowing fusion between viral and host cellular membranes, resulting in the release of viral RNA into the cell cytoplasm [27]. TMPRSS2 and DPP4 are held in one complex at the cell surface by a scaffolding protein, the tetraspanin CD9, leading to a rapid and efficient entry of MERS-CoV into the susceptible cells [43]. Once fusion with host cell membranes has occurred, MERS-CoV subsequently replicates its genetic material and produces viral proteins in the cell cytoplasm to generate new virus progeny. During this stage, MERS-CoV uses its nsp3–4 polyproteins to build its replication organelles as well as its accessory proteins such as the 4a and 4b proteins to inhibit host anti-viral defense mechanisms [44,45,46,47,48,49,50,51,52,53,54]. However, the capacity of MERS-CoV accessory proteins to impede several pathways of host immune response in the lungs may be limited. MERS-CoV inoculation of macaques and genetically modified mice generally results in limited clinical manifestations; thus, adapting this virus through serial passaging or defecting the type I interferon pathway may be needed to enhance viral replication and pathogenesis in these animals [32,55,56,57,58]. These observations, together with studies showing type I interferon capacity to inhibit MERS-CoV infection in vitro [25,59], highlight the importance of the innate immune response, especially type I interferon, as an inhibiting factor for MERS-CoV.

## 3. Host Factors in MERS-CoV Transmission

So far MERS-CoV has been isolated from dromedary camels and humans [1,60]. Both species are not only susceptible to MERS-CoV infection, but also capable of transmitting this virus [7,12,13,14,15,16,17,18,22]. However, current data indicate that virus spread is more efficient in dromedary camels than in humans [5,7,19,20,21,61]. This difference in transmissibility could be partially due to the different tropism of MERS-CoV in these two species. In dromedaries, MERS-CoV has been shown to replicate in the nasal epithelium upon experimental in vivo infection [17], while in humans, MERS-CoV mainly replicates in the lower respiratory tract, particularly in the bronchiolar and alveolar epithelia [23,62,63,64,65]. Higher viral RNA levels in the sputum and lavage samples of MERS-CoV patients compared to nasal and throat swabs are consistent with the tropism of MERS-CoV in humans [66,67,68]. This different MERS-CoV tropism in dromedary camels and humans is in line with the localization of DPP4 in the respiratory tract tissues of these two species. In humans, DPP4 is absent in the nasal epithelium but present in the lower respiratory tract epithelium, mainly in type II pneumocytes [69,70]. In contrast, DPP4 is expressed in the nasal epithelium of dromedary camels [69]. This difference in DPP4 localization between humans and dromedary camels therefore explains MERS-CoV tropism in these two species and highlights DPP4 as an essential determinant of MERS-CoV tropism.

DPP4 localization has also been investigated in many other MERS-CoV-susceptible species. In Gambian and Egyptian fruit bats, DPP4 is expressed in the respiratory tract and intestinal epithelium, suggesting that MERS-CoV can target both tissues [71]. In line with this finding, MERS-CoV inoculation via intranasal and intraperitoneal routes in the Jamaican fruit bat led to viral RNA shedding both in the respiratory tract and the intestinal tract [72]. In contrast to frugivorous bats, DPP4 is limitedly expressed in the respiratory tract epithelium of two insectivorous bats, i.e., common pipistrelle and common serotine bats, but abundant in their intestinal epithelium [71]. Accordingly, sequences of MERS-like-CoVs were mainly obtained from rectal swabs and fecal samples of insectivorous bats [73,74,75,76,77,78,79,80]. These findings not only support insectivorous bats as the origin host of MERS-CoV [73,74,75,76,77,78,79,80], but also indicate the importance of intestinal tropism and fecal–oral transmission of MERS-like-CoV in these insectivorous bats.

Besides bats, humans, and dromedary camels, other animal species have also been proposed as potential hosts of MERS-CoV. Remarkably, DPP4 of horses, llamas, alpacas, pigs, bovines, goats, sheep, and rabbits has been demonstrated to recognize the S protein of MERS-CoV [81,82]. In most of these species, there is a preferential upper respiratory tract expression of DPP4 observed. Rabbits express DPP4 in the upper and lower respiratory tract epithelium, and thus may allow MERS-CoV to replicate in both compartments [33,83]. Horses, llamas, and pigs mainly express DPP4 in the upper respiratory tract—particularly the nasal epithelium [84]. Upon intranasal MERS-CoV inoculation, llamas, alpacas, and pigs developed upper respiratory tract infection, while horses did not seroconvert and only shed infectious virus in a limited amount [84,85,86,87,88]. The reason why horses seem to be less permissive to MERS-CoV remains to be investigated, but a chronic co-infection in the guttural pouch, a common disease among horses, might be one of the explanations. This guttural pouch infection results in excessive mucus production that might hinder MERS-CoV from attaching and entering the nasal epithelium [84,89,90]. Sheep, on the other hand, did not seem to express significant levels of DPP4 in their respiratory tract, and thus did not seroconvert nor shed infectious virus upon experimental MERS-CoV inoculation [84,88]. Comparable to sheep, goats limitedly shed infectious virus upon experimental infection and did not transmit this virus to other naïve goats upon direct contact [88]. The results of experimental MERS-CoV infection in livestock animals are in line with data from epidemiological studies. MERS-CoV seropositive llamas and alpacas are present in the field, while horses, goats, and sheep are generally found to be seronegative [22,86,87,91,92,93,94,95,96,97,98].

**Figure 2:**
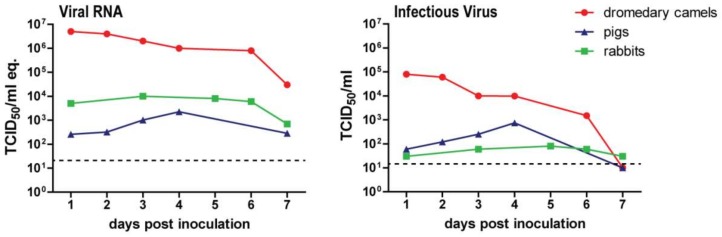
Schematic overview of viral RNA and infectious virus shedding of MERS-CoV-inoculated dromedary camels, pigs, and rabbits. Each data point represents the average data from previous experiments [17,33,84]. Viral RNA is measured in TCID_50_/mL genome equivalents, while infectious virus is expressed in TCID_50_/mL.

**Figure 3:**
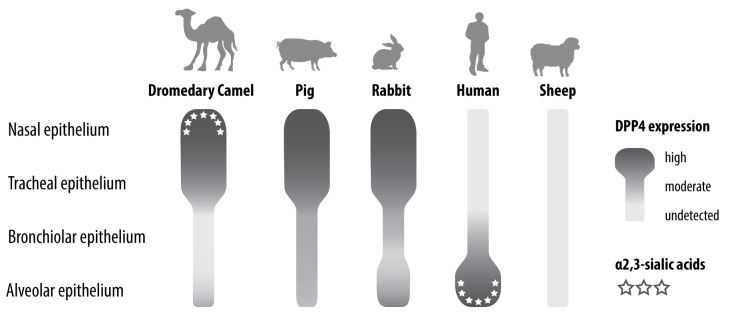
Schematic representation of DPP4 expression and MERS-CoV-recognized α2,3-sialic acid glycotopes in the respiratory tract of dromedary camel, pig, rabbit, human, and sheep.

Given the fact that experimental in vivo infection studies and DPP4 expression analysis in different animal species revealed that dromedary camels are not the only animals in which MERS-CoV has an upper respiratory tract tropism [17,18,83,84], it is then relevant to question whether other animals can potentially spread MERS-CoV as well. New World camelids, i.e., alpacas and llamas, are able to transmit the virus to respective naïve animals upon contact [86]. Pigs and rabbits, on the other hand, hardly transmit the virus—neither by contact nor airborne routes [83,99]. Most likely, this is caused by the fact that pigs and rabbits, unlike dromedary camels, shed low levels of infectious virus upon MERS-CoV inoculation (Figure 2). This difference indicates that other host factors besides DPP4 could cause interspecies variation in MERS-CoV infection. Indeed, several glycotopes of α2,3-sialic acids that function as attachment factors of MERS-CoV are present in the nasal epithelium of dromedary camels but absent in that of rabbits and pigs (Figure 3) [24,100]. The lack of these glycotopes in pigs and rabbits might limit the susceptibility and transmission of MERS-CoV in these animals. Although the role of these glycotopes in MERS-CoV transmission still requires further investigation, it remains plausible that an efficient transmission of this virus might require the presence of both DPP4 and MERS-CoV-recognized glycotopes of α2,3-sialic acids (Figure 3).

Besides entry and attachment receptors, MERS-CoV has been demonstrated to use both cell surface and lysosomal proteases to enter its target cells [39,40,43,101]. The preference of MERS-CoV to use certain host proteases is influenced by the type of target cell and the cleavage stage of their S protein prior to infection [40]. It has also been reported that the lysosomal proteases from bat cells support coronavirus spike-mediated virus entry more efficiently than their counterparts from human cells [39]. These observations suggest that host proteases from different host species may determine the species and tissue tropism of MERS-CoV.

Because MERS-CoV has been circulating in dromedary camels for decades before emerging in the human population [19,20,21,22], it is plausible that this virus inhibits the immune response of dromedary camels more efficiently than that of other species, including pigs and rabbits. The difference in immune response among MERS-CoV-susceptible species is therefore another factor that might yield interspecies variation in permissiveness to MERS-CoV. Characterizing the difference in host proteases and immune responses among MERS-CoV-susceptible species, as performed for DPP4 and MERS-CoV-recognized α2,3-sialic acid glycotopes (Figure 3), has not yet been investigated. These data, however, may further explain interspecies variation in MERS-CoV infection and transmission.

## 4. Host Factors in MERS-CoV Pathogenesis

MERS-CoV causes respiratory infection in humans ranging from asymptomatic to severe pneumonia [3,4]. However, it is currently unclear what causes this intraspecies variation. Epidemiology data indicate that individuals with certain risk factors are at higher risk of developing severe MERS-CoV infection [4,102]. This implies that some host factors may dictate the outcome of MERS-CoV infection, thus rendering intraspecies variation. Two of the risk factors, i.e., smoking and chronic obstructive pulmonary disease (COPD), have been shown to upregulate DPP4 expression in the lungs [70,102,103,104], suggesting DPP4 as a possible reason for intraspecies variation observed among MERS-CoV patients. In healthy human lungs, DPP4 is almost exclusively expressed in type II pneumocytes [69,70]. Type II pneumocytes are small cuboidal cells that can regenerate alveolar epithelium upon injury, and roughly cover 2% of the alveolar surface area. Meanwhile, around 95% of the surface area of the alveolus is occupied by type I pneumocytes that are morphologically flat and responsible for gas exchange [105,106]. In the lungs of smokers and COPD patients, unlike in healthy human lungs, DPP4 is prominently expressed in both type I and II pneumocytes, indicating upregulated expression on type I pneumocytes [104]. Autopsy reports from fatal MERS-CoV patients showed that both type I and II pneumocytes expressed DPP4 and became infected by MERS-CoV, proposing a role of DPP4-expressing type I pneumocytes in MERS-CoV pathogenesis [64,107]. Damage to type I cells in the lung alveoli during viral infection may lead to diffuse alveolar damage [108]. In line with observations made in human MERS cases, common marmosets that express DPP4 in both type I and II pneumocytes have been reported to produce more infectious virus upon experimental MERS-CoV infection, compared to rhesus and cynomolgus macaques that merely expressed DPP4 in type II pneumocytes [58,109,110,111,112]. Accordingly, these common marmosets developed moderate-to-severe infection, while macaques generally developed mild transient pneumonia [32,58,109,110,111,112]. Similarly, in genetically modified mice that displayed MERS-CoV tropism for type II pneumocytes, only mild clinical manifestations were observed upon MERS-CoV infection [56,113]. Adapting MERS-CoV through serial passaging or upregulating DPP4 expression throughout the airway epithelium in mice, however, will induce severe clinical disease [55,56]. These data altogether support the role of DPP4-expressing type I pneumocytes in the pathogenesis of severe MERS-CoV infection.

The differential expression of host factors that limits the infection should also be taken into account. DPP4 in soluble form has been demonstrated to protect against MERS-CoV infection in vitro and in a mouse model [23,114]; however, its presence in the lungs and role in MERS-CoV pathogenesis remain to be investigated. The host immune response also has the capacity to inhibit MERS-CoV infection. MERS-CoV has been shown to replicate to higher levels in immunocompromised rhesus macaques [115], consistent with the observation that immunocompromised individuals have difficulties clearing MERS-CoV upon infection [68,107,116]. The survivors of MERS-CoV infection have been shown to develop virus-specific CD4+ and CD8+ T cell responses, implying the role of T cells in virus clearance [117]. However, the depletion of T cells in mice can either lead to failure in MERS-CoV clearance or improvement in clinical outcome, depending on the type of mouse model used [57,118]. Therefore, the role of adaptive immune response in MERS-CoV pathogenesis is currently unclear. On the other hand, one of the main components of the host innate immune response, type I interferon, inhibits MERS-CoV replication in susceptible cells, partly by inhibiting double membrane vesicles (DMV) formation [25,57,59,119,120]. The absence of type I interferon signaling in mice also resulted in more severe clinical manifestations and histopathological lesions upon MERS-CoV infection [57]. Advance age, which can cause delayed type I interferon response upon viral infection, is a well-known risk factor for fatal MERS-CoV infection [4,102,121,122,123]. Collectively, these data highlight the role of host innate immune response as a potent inhibitor for MERS-CoV infection.

**Figure 4:**
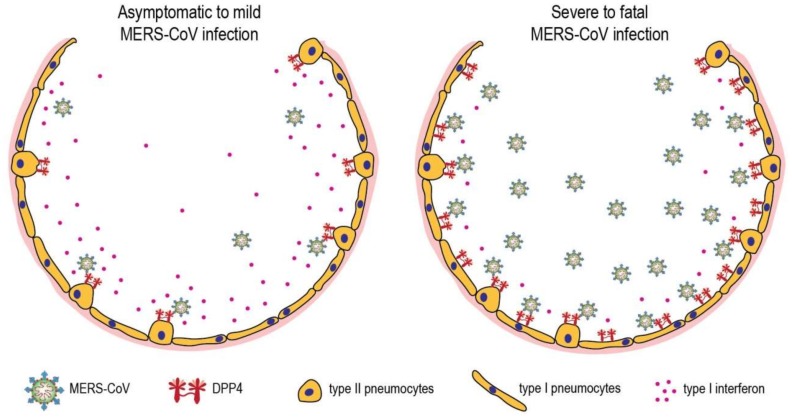
MERS-CoV infection in the lungs of asymptomatic-to-mild (left panel) and severe-to-fatal cases (right panel). Shown is a hypothetical model with two critical host determinants, DPP4 and interferon, differentially expressed in asymptomatic-to-mild and severe-to-fatal MERS-CoV infection.

It is indubitable that severe MERS-CoV infection is not solely driven by the pathogen. Additional underlying conditions increase MERS-CoV replication and induce severe-to-fatal clinical manifestations [4,11,103,124,125]. It is plausible that more than one underlying condition is needed to yield a fatal outcome. DPP4 upregulation in type I pneumocytes and insufficient type I interferon response might be crucial determinants for severe MERS-CoV infection (Figure 4). Further investigation of the host determinants of MERS-CoV pathogenesis may offer insights for developing novel therapeutic measures.

## 5. Concluding Remarks and Future Perspectives

Although MERS-CoV has been reported to undergo some genotypic changes since it emerged in the human population [12,126,127,128,129], this has not resulted in distinct phenotypic changes so far [63,126]. Therefore, host factors remain the most significant determinant in explaining inter- and intraspecies variations observed in MERS-CoV pathogenesis and transmission. DPP4 and MERS-CoV-recognized α2,3-sialic acids might partially explain these variations, since their localization has been demonstrated to be variable between MERS-CoV-susceptible species [69,71,84,100]. DPP4 expression in human lungs has also been shown to vary due to certain comorbidities [70,96,104]. Nevertheless, it is undoubtable that the inter- and intraspecies variation in MERS-CoV pathogenesis and transmission is a complex phenomenon influenced by more than one host factor. Current data suggest proteases and interferons as other critical host factors, but how they instigate inter- and intraspecies variations, as well as their role in MERS-CoV pathogenesis and transmission, still remain to be further elucidated. Characterization of the host determinants of MERS-CoV pathogenesis and transmission could potentially offer insight into this virus epidemiology and guide novel therapeutic development. It may also help to identify the most vulnerable individuals to protect against MERS-CoV infection—for example, by using vaccination.
